# Diagnostic Significance of Plasma Levels of Novel Adipokines in Patients With Symptomatic Intra- and Extracranial Atherosclerotic Stenosis

**DOI:** 10.3389/fneur.2019.01228

**Published:** 2019-11-19

**Authors:** Fang Yu, Xiaoqing Zhou, Zhibin Li, Xianjing Feng, Di Liao, Zeyu Liu, Qin Huang, Xi Li, Qidong Yang, Bo Xiao, Jian Xia

**Affiliations:** ^1^Department of Neurology, Xiangya Hospital, Central South University, Changsha, China; ^2^Hunan Clinical Research Center for Cerebrovascular Disease, Changsha, China

**Keywords:** symptomatic intracranial atherosclerotic stenosis, symptomatic extracranial atherosclerotic stenosis, apelin, omentin, visfatin, RBP-4

## Abstract

**Background:** Adipokines have been proven to be associated with atherosclerotic diseases such as ischemic stroke and coronary heart disease. The role of novel adipokines in the development of symptomatic intracranial atherosclerotic stenosis (sICAS) and extracranial atherosclerotic stenosis (sECAS) has not yet been investigated. This study aimed to evaluate the plasma levels of novel adipokines in patients with sICAS and sECAS and their associations with the prognosis of sICAS groups.

**Methods:** A total of 134 patients with acute ischemic stroke attribute to large-artery atherosclerosis (LAA) and 66 age- and sex-matched controls without atherosclerotic stenosis (NCAS) were included in this study. The LAA group was further sub-classified as sICAS (*n* = 102) and sECAS (*n* = 32) according to the location of atherosclerosis. Demographics, clinical parameters, angiographical features and plasma levels of novel adipokines (apelin, visfatin, omentin, RBP-4) were assayed and compared among groups.

**Results:** LAA patients had significantly lower levels of omentin [39.92 (30.74–52.61) ng/ml vs. 54.42 (34.73–79.91) ng/ml, *P* < 0.001] and visfatin [11.32 (7.62–16.44) ng/ml vs. 13.01 (9.46–27.54) ng/ml, *P* < 0.001] than those in the NCAS group. Multiple logistic regression analysis identified that the lowest tertile of omentin was independently associated with LAA (OR, 3.423; 95% CI, 1.267–9.244, when referenced to the third tertile). Levels of omentin, visfatin and RBP-4 showed no significant difference between sICAS and sECAS groups. However, median concentrations of apelin were lower in sECAS [84.94 (46.88–130.41) ng/mL) than in sICAS [118.64 (93.22–145.08) ng/mL, *P* = 0.002] and NCAS [114.38 (80.56–162.93) ng/mL, *P* = 0.004]. Logistic regression analysis showed that the lowermost tertile of apelin was independently associated with sECAS (OR, 5.121; 95% CI, 1.597–16.426) when adjusted for risk factors. As for sICAS patients, spearman coefficient analysis showed no significant correlation between these four adipokines and the severity of sICAS or the number of vessels with intracranial stenoses. Patients with severe stroke had lower levels of apelin (*P* = 0.005), while the other three adipokines showed no such difference. During follow up, no difference was found between these four novel adipokines and short- and long-term outcome of sICAS.

**Conclusions:** Lower levels of omentin are independent biomarkers of LAA while low apelin plasma levels seem to be risk factors of sECAS.

## Introduction

Large-artery atherosclerosis (LAA) stroke is a highly prevalent cause of cerebral infarction according to the classification standard of Trial of Org 10172 in Acute Stroke Treatment (1). LAA affects both intracranial and extracranial arteries and symptomatic intracranial atherosclerotic stenosis (sICAS) has been proposed to be the major cause of LAA among Asian and African patients. On the contrary, symptomatic extracranial atherosclerotic stenosis (sECAS) is more common in Caucasians (2, 3). sICAS is a developing and progressing disease; sICAS patients are at high risk of stroke recurrence and developing other vascular events like myocardial infarction and peripheral vascular disease (4). Even under aggressive medical treatment and risk factor mitigation, sICAS-related recurrence risk continues to be high (5). Given the large burden of post-sICAS, assessment of the presence and prognosis of this disease is crucial. The potential pathogenesis of sICAS and the underlying causes behind the difference between intra- and extracranial stenosis remain unknown. Pathophysiological mechanisms like endothelial injury, lipid deposition, inflammation, angiogenesis and impaired fibrinolysis, etc. may play important roles in the pathological process of sICAS (6–9). Conventional risk factors like smoking, hypertension (HBP), diabetes mellitus (DM), and metabolic syndrome (MetS) have been reported to contribute differently to the development of sICAS and sECAS (7, 10–13).

Several studies have investigated the diagnostic or prognostic value of biomarkers like matrix metalloproteinases (MMP-2), Lipoprotein-associated phospholipase A2 (Lp-PLA2), Vascular Endothelial Growth Factor (VEGF) and Endostatin in patients with sICAS or sECAS (5, 7, 14). However, some conclusions have failed to be validated for these studies. Adipose tissue is an endocrine organ which can produce several adipokines that can modulate insulin sensitivity and contribute to the pathogenesis of MetS, DM, dyslipidemia, inflammation, endothelial dysfunction, coronary artery disease (CAD), and atherosclerotic stroke (15, 16). Adiponectin, leptin, and resistin are the most well-studied adipokines. The role of these traditional adipokines in cerebral vascular atherosclerotic stenosis has been reported in several studies. O.Y. Bang reported that lower adiponectin levels were associated with symptomatic sICAS vs. other ischemic stroke subtypes (17). Another study from South Korea showed that lower levels of adiponectin and higher levels of leptin had significant associations with increased risk of LAA stroke, but not sICAS (18). The Barcelona-AsIA (Asymptomatic Intracranial Atherosclerosis) Study suggested that resistin was independently associated with combined intracranial-extracranial atherosclerotic disease (19).

Emerging evidence suggests a relationship between novel adipokines such as apelin, omentin, visfatin, RBP-4, and vascular risk factors and atherosclerotic diseases. Apelin, encoded by APLN gene, is a peptide that functions as an endogenous ligand of the orphan G-protein-coupled receptor. Apelin serum levels have been found to be increased in diabetic patients and in patients with metabolic syndrome and have been linked with coronary atherosclerosis (20, 21). On the contrary, Kadoglou et al. has supported an inverse relationship between serum apelin concentration and carotid atherosclerosis (22). Omentin, encoded by ITLN1 gene, is a novel adipokine produced by visceral adipose tissue. Omentin has been intimately linked with protective mechanisms against insulin-resistant states, for example, obesity and diabetes (23). Moreover, low omentin-1 levels have been detected in patients with carotid atherosclerosis and ischemic stroke (24, 25), and higher levels of omentin-1 have been associated with poor functional outcome of acute ischemic stroke (IS) patients (26). Visfatin, mainly secreted by visceral adipose tissue, is also known as nicotinamide phosphoribosyl transferase (NAMPT). Elevated visfatin levels have been found in DM, obesity, CAD, and symptomatic carotid stenosis (27–30). Another newly identified adipokine, Retinol-binding protein 4 (RBP-4), has emerged as a possible regulator of insulin resistance, obesity, and inflammation (31). Studies have shown that RBP-4 is positively correlated with the presence of CAD and carotid atherosclerosis (24, 32). To our knowledge, no study has examined the relationship of these four novel adipokines with sICAS or focused on their different roles in sICAS and sECAS. Given the relationship between the four novel adipokines with obesity, diabetes, inflammation, and atherosclerosis, we can infer that they may have additional connections with sICAS.

The purpose of our research was to study the association between plasma levels of four novel adipokines (apelin, visfatin, omentin, RBP-4) in sICAS and sECAS patients. We also examined the relationship between the plasma levels of these adipokines and the prognosis of sICAS patients.

## Patients and Methods

### Study Population

**Figure 1:**
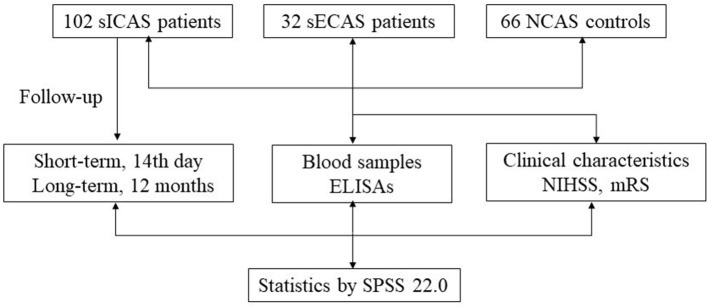
Study flow chart. sICAS, symptomatic intracranial atherosclerotic stenosis disease; sECAS, symptomatic extracranial atherosclerotic stenosis disease; NCAS, no atherosclerotic stenosis controls; NIHSS, National Institutes of Health Stroke Scale; mRS, modified Rankin Scale; ELISA, enzyme-linked immunosorbent assay.

Study flow chart is shown in Figure 1. We prospectively recruited patients with acute large-artery atherosclerotic stroke (<7 days of onset) with relevant neuroimaging findings from September 2015 to May 2017 in the Department of Neurology, Xiangya Hospital. At the same time, 66 controls without atherosclerotic stenosis (NCAS) who were age- and sex-matched from the Health Examination Center of Xiangya Hospital were included. The exclusion criteria were as follows: infection on admission, autoimmune rheumatic diseases, malignancies, renal and hepatic insufficiency, recent surgery and those who underwent incomplete vascular imaging and laboratory tests, those who had stroke of other determined etiologies and those who did not provide informed consent. Patients who had combined intracranial and extracranial artery stenoses were also excluded. All subjects signed the informed consent and the study protocol was approved by the Ethics Committee of Xiangya Hospital of the Central South University in China.

### Imaging Assessment

Using our previously published angiographic assessment (7, 33), all patients underwent Brain Magnetic Resonance Imaging (MRI) scans to determine whether cerebral infarction exists. Most patients completed Magnetic Resonance Angiography (MRA) examination on a 1.5 or 3.0 T magnetic resonance scanner and CT angiography was performed in patients with contraindication of MRI. All patients underwent evaluations of extracranial vessels like carotid-enhanced magnetic resonance angiography (CE-MRA), carotid CTA, and ultrasonography of cervical vessels. Digital subtraction angiography (DSA) and high-resolution magnetic resonance imaging (HR-MRI) were used to validate the results of MRA/CEMRA/CTA findings. Intracranial vessels are defined as the proximal portion of internal carotid artery (ICA) and vertebral artery (VA), basilar artery (BA), anterior cerebral artery (ACA), middle cerebral artery (MCA), and posterior cerebral artery (PCA). Extracranial vessels are the extra-segment of ICA and VA. Intracranial artery stenosis was evaluated by referring to the published method in Warfarin–Aspirin Symptomatic Intracranial Disease Study (34). Stenosis degree of extracranial ICA and VA was calculated by the formula published by Rothwell et al. (35). The number of stenotic arteries of each patient was also included. According to stenosis severity, sICAS were categorized into moderate (50–69%) and severe (≥70%) groups. Finally, 102 patients with sICAS and 32 patients with sECAS were included in this study. Combined cerebral atherosclerotic stenosis (CCAS) was excluded from this study because of the small number of cases.

### Clinical Assessment

Traditional risk factors like HBP, DM, dyslipidemia, CAD, smoking, and drinking were collected by clinical questionnaire and were defined as previously published (7, 36–38). Additionally, we also collected data on demographics, blood pressure, FBG (fasting glucose levels), HbA1C (glycated hemoglobin), TC (total cholesterol) levels, LDL (low-density lipoprotein cholesterol) levels, HDL (high-density lipoprotein cholesterol) levels, and TG (triglyceride) levels. All venous blood samples were drawn at baseline after an overnight fast at least for 12 h, the median time of blood sampling was 4 days after stroke onset. Metabolic syndrome was defined as having at least three conditions: men with waist-circumference ≥90 cm or women ≥80 cm; triglycerides >1.7 mmol/L; men with HDL cholesterol <1.03 mmol/L or women <1.30 mmol/L; already taking antihypertensive drugs or blood pressure ≥130/85 mmHg; previously diagnosed with type 2 DM, or taking antidiabetic drugs or current fasting blood glucose ≥5.6 mmol/L (39).

At admission, the National Institutes of Health Stroke Scale (NIHSS) was evaluated to define stroke severity. NIHSS ≤ 8 was defined as mild stroke and >8 was defined as severe stroke. The short-term outcome was measured on the 14th day after stroke according to the modified Rankin Scale (mRS) score. mRS ≤ 2 was defined as favorable outcome and mRS >2 or death was defined as unfavorable outcome. Then all patients were followed-up with 12 months after the discharge, information was obtained by telephone interview.

### Determination of Novel Adipokines in Plasma

EDTA tubes were used to collect the venous blood samples at baseline. The median time of blood sampling was 4 days after stroke onset. The blood samples were centrifuged at 3,000 rpm at 4°Cfor 15 min immediately after collection. We collected plasma samples and stored them in an −80°C ultra-low temperature refrigerator until analysis. Plasma concentrations of apelin, omentin, visfatin, and RBP-4 (RayBiotech, Inc., Norcross, GA, USA) were assayed using quantitative sandwich enzyme immunoassay commercially available kits. ELISAs were performed according to the RayBio® ELISA manual strictly. All samples were assayed twice. The intra-assay coefficients of variance (CVs) were <10% for apelin, omentin, visfatin and RBP-4, while the inter-assay CVs for these variables were <15% for apelin, omentin, visfatin and <12% for RBP-4. All absorption readings were performed according to the standard curve.

### Statistical Analysis

SPSS 22.0 (IBM SPSS, Chicago, Ill, USA) was used for all statistical analysis. Kolmogorov-Smirnov test was used to evaluate the normality of distribution. Normal distribution data were expressed as mean ± SD, and non-normal distribution data were expressed as median and quartile range. Adipokine levels were not normally distributed in our study. Category variables were expressed as percentages. Classified variables were tested by χ^2^-test and Fisher exact test to evaluate the statistical significance of differences between groups. One-way ANOVA and independent sample *t*-test were used to compare the parameters of normal distribution. Kruskal-Wallis test and Mann-Whitney *U*-test were used to compare the variables of non-normal distribution. Spearman correlation coefficient was used to study the correlation between the number and severity of sICAS and adipokines levels. In further analysis, adipokines levels were divided into several groups according to tertiles. Variables showing *P* < 0.05 in the univariate analysis and potential confounding factors such as age, gender, hypertension, diabetes, dyslipidemia, and MetS were included in the logistic regression analysis. Continuous variables were converted into categorized variables to get better and more reasonable interpretations of the results. The mRS at 14th days was not included as covariates in the regression analysis because it was the definition of early functional outcome, not the risk factors. The accuracy of biomarkers in disease prediction were measured by receiver operating characteristics (ROC) curves. Due to the small sample size of sECAS, biomarkers did not allow reliable analyses by subgroups. As for multiple comparisons, the significance level was adjusted to 0.05/4 ≈ 0.0125 with the Bonferroni method.

## Results

### Study Population Characteristics

A total of 134 patients with LAA and 66 age and sex-matching NCAS controls were included in our study. The LAA group was further subclassified into 102 patients with sICAS and 32 with sECAS. Baseline demographic data, vascular risk factors, and laboratory test results of study participants are depicted in Table 1. Among all individuals, 149 (74.5%) had hypertension, 55 (27.5%) had diabetes mellitus, 103 (51.5%) had dyslipidemia, and 33 (16.5%) had coronary heart disease. Seventy-eight (39.0%) patients were smokers and 53 (26.5%) patients were drinkers. Median plasma levels of apelin, omentin, visfatin, and RBP-4 were 114.69, 43.25, 12.08 ng/mL, and 76.19 mg/L, respectively.

**Table 1:** Baseline characteristics and adipokines concentrations among groups.

	**LAA (*n* = 134)**	**sICAS (*n* = 102)**	**sECAS (*n* = 32)**	**NCAS (*n* = 66)**	***P*-value^a^**	***P*-value^b^**	***P*-value^c^**	***P*-value^d^**
Age (years)	62 (52–69)	63 (56–69)	61 (51–70)	61 (53–67)	0.401	0.326	0.876	0.575
Male [*n* (%)]	87 (64.9%)	60 (58.8%)	27 (84.4%)	35 (53.0%)	0.124	0.459	0.003	0.010
Hypertension [*n* (%)]	110 (82.1%)	86 (84.3%)	24 (75.0%)	39 (59.1%)	0.001	<0.001	0.123	0.290
SBP (mmHg)	144 (133–156)	146 (136–156)	142 (128–151)	133 (118–150)	0.002	0.001	0.194	0.254
DBP (mmHg)	84 (76–95)	85 (77–95)	82 (70–94)	80 (71–88)	0.009	0.003	0.471	0.132
Diabetes [*n* (%)]	38 (28.4%)	30 (29.4%)	8 (25.0%)	17 (25.8%)	0.739	0.918	0.376	0.822
FPG (mmol/L)	5.34 (4.79–6.53)	5.31 (4.79–6.39)	5.97 (4.59–7.47)	5.85 (5.11–5.85)	0.035	0.015	0.654	0.429
HbA1C (%)	5.90 (5.53–6.60)	5.90 (5.50–6.60)	5.90 (5.60–6.70)	5.70 (5.50–6.20)	0.192	0.263	0.234	0.767
Dyslipidemia [*n* (%)]	79 (59.0%)	64 (62.7%)	15 (46.9%)	24 (36.4%)	0.004	0.001	0.319	0.149
TG (mmol/L)	1.76 (1.14–2.36)	1.79 (1.14–2.45)	1.68 (1.16–2.29)	1.47 (1.13–1.96)	0.081	0.076	0.344	0.550
TC (mmol/L)	4.73 ± 1.20	4.74 ± 1.20	4.71 ± 1.23	4.99 ± 1.06	0.135	0.165	0.238	0.899
LDL (mmol/L)	2.94 ± 0.95	2.96 ± 0.94	2.89 ± 0.99	2.97 ± 0.84	0.858	0.952	0.699	0.736
HDL (mmol/L)	1.18 (0.96–1.40)	1.18 (0.95–1.39)	1.16 (1.06–1.40)	1.39 (1.18–1.57)	<0.001	<0.001	0.011	0.383
CAD [*n* (%)]	20 (14.9%)	16 (15.7%)	4 (12.5%)	13 (19.7%)	0.421	0.502	0.378	0.782
MetS [*n* (%)]	50 (37.3%)	40 (39.2%)	10 (31.3%)	20 (30.3%)	0.349	0.239	0.924	0.531
Smoking [*n* (%)]	56 (41.8%)	41 (40.2%)	15 (46.9%)	22 (33.3%)	0.282	0.370	0.195	0.542
Drinking [*n* (%)]	39 (29.1%)	26 (25.5%)	13 (40.6%)	14 (21.2%)	0.307	0.525	0.044	0.120
Hospital stays	14 (10–16)	13 (9–16)	14 (11–20)	–	-	–	–	0.213
NIHSS on admission	4 (2–8)	5 (3–8)	3 (1–7)	–	-	–	–	0.036
mRS at 14th days	2(1–4)	3 (1–4)	1 (0–3)	–	–	–	–	0.007
Adipokines								
Apelin (ng/ml)	115.08 (85.16–142.75)	118.64 (93.22–145.08)	84.94 (46.88–130.41)	114.38 (80.56–162.93)	0.310	0.982	0.004	0.002
Omentin (ng/ml)	39.92 (30.74–52.61)	39.43 (29.86–51.21)	40.54 (31.29–55.25)	54.42 (34.73–79.91)	<0.001	<0.001	0.022	0.748
Visfatin (ng/ml)	11.32 (7.62–16.44)	11.93 (6.13–16.72)	10.38 (8.02–14.36)	13.01 (9.46–27.54)	0.001	0.002	0.006	0.561
RBP-4 (mg/L)	76.69 (50.66–109.64)	76.07 (52.40–109.64)	77.26 (38.59–107.23)	75.54 (36.40–125.25)	0.601	0.490	0.940	0.637

*LAA, large-artery atherosclerosis; sICAS, symptomatic intracranial atherosclerotic stenosis disease; sECAS, symptomatic extracranial atherosclerotic stenosis disease; NCAS, no atherosclerotic stenosis controls; SBP, systolic blood pressure; DBP, diastolic blood pressure; FBG, fasting plasma glucose; HbA1C, glycated hemoglobin; TG, triglyceride; TC, total cholesterol; LDL, low density lipoprotein cholesterol; HDL, high density lipoprotein cholesterol; CAD, coronary heart disease; Mets: metabolic syndrome; BMI: Body Mass Index; NIHSS, the National Institutes of Health Stroke Scale; mRS, the modified Rankins Score*.

a *P-value: LAA vs. NCAS*.

b *P-value: sICAS vs. NCAS*.

c *P-value: sECAS vs. NCAS*.

d *P-value: sICAS vs. sECAS*.

### Plasma Adipokines Between Groups

**Figure 2:**
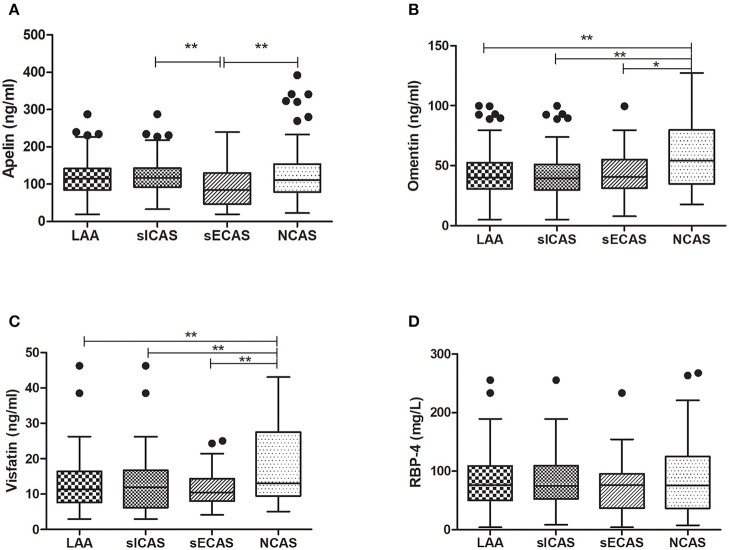
Levels of plasma adipokines in different groups. *P < 0.05 and **P < 0.01. *n* = 134 in LAA, *n* = 102 in sICAS, *n* = 32 in sECAS, *n* = 66 in NCAS. LAA, large-artery atherosclerosis; sICAS, symptomatic intracranial atherosclerotic stenosis disease; sECAS, symptomatic extracranial atherosclerotic stenosis disease; NCAS, no atherosclerotic stenosis controls.

**Figure 3:**
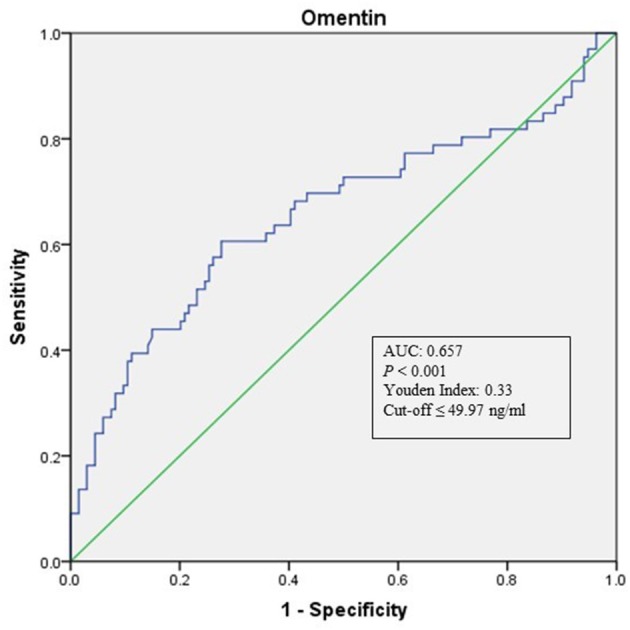
ROC curve analysis of Omentin cutoff point for the presence of LAA (vs. NCAS). AUC, area under the curve; ROC, receiver operating characteristic; LAA, large-artery atherosclerosis; NCAS, no atherosclerotic stenosis controls.

As shown in Table 1, when compared with NCAS controls, patients with LAA had significantly decreased levels of HDL and higher levels of SBP and DBP (*P* < 0.0125, Bonferroni correction). Patients with LAA showed higher frequency of hypertension and dyslipidemia than individuals in the control group. Moreover, the LAA group displayed lower plasma concentrations of omentin [39.92 (30.74–52.61) ng/ml vs. 54.42 (34.73–79.91) ng/ml, *P* < 0.001] and visfatin [11.32 (7.62–16.44) ng/ml vs. 13.01 (9.46–27.54) ng/ml, *P* = 0.001] than control counterparts, while the two study groups had non-significant differences between apelin and RBP-4 levels (shown in Figure 2). The levels of omentin and visfatin were then grouped according to tertiles. Variables showing *P* < 0.05 in the univariate analysis were selected for entry into logistic regression analysis. And we also adjusted with age, gender (male vs. female), hypertension, diabetes, dyslipidemia, and MetS. Logistic regression analysis revealed that the lowest tertile of omentin was independently associated with LAA (OR, 3.423; 95% CI, 1.267–9.244, when referenced to the third tertile). Tertiles of visfatin showed no significance when adjusted with their confounding factors (as shown in Table 2). With ROC curves analysis, omentin had reasonable accuracy for prediction of LAA (AUC: 0.657, Youden Index: 0.33, *P* < 0.001). The optimal cut-off value of omentin was ≤ 49.97 ng/mL (sensitivity: 0.606; specificity: 0.724) (as Figure 3 showed).

**Table 2:** Logistic regression analysis with the presence of LAA as the dependent variable.

	**LAA vs. NCAS**		
	***P-*value**	**OR**	**95% CI for OR**
Age	0.447	1.014	0.978–1.051
Gender (Male vs. Female)	0.154	1.706	0.818–3.560
Hypertension	0.042	2.706	1.039–7.046
Diabetes	0.268	1.925	0.604–6.135
Dyslipidemia	0.095	1.964	0.890–4.335
MetS	0.420	0.650	0.228–1.851
SBP	0.125	1.019	0.995–1.043
DBP	0.672	0.992	0.959–1.028
FBG	0.024	0.795	0.651–0.970
HDL	0.001	0.147	0.048–0.448
Omentin tertiles, ng/ml			
1 (<34.44)	0.015	3.423	1.267–9.244
2 (34.44–53.24)	0.062	2.502	0.956–6.544
3 (>53.24)	Reference	-	-
*P* for trend = 0.043			
Visfatin tertiles, ng/ml			
1 (<9.60)	0.784	1.155	0.413–3.232
2 (9.60–15.28)	0.864	0.921	0.358–2.370
3 (>15.28)	Reference	-	-
*P* for trend = 0.839			

*LAA, large-artery atherosclerosis; NCAS, no atherosclerotic stenosis controls; SBP, systolic blood pressure; DBP, diastolic blood pressure; FBG, fasting plasma glucose; HDL, high density lipoprotein cholesterol; Mets, metabolic syndrome. NCAS is as reference*.

When compared with NCAS controls, patients with sICAS had significantly decreased levels of HDL and higher levels of SBP and DBP (*P* < 0.0125, Bonferroni correction). Patients with sICAS showed higher frequency of hypertension and dyslipidemia than control individuals. Moreover, sICAS patients appeared to have lower plasma concentrations of omentin [39.43 (29.86–51.21) ng/ml vs. 54.42 (34.73–79.91) ng/ml, *P* < 0.001] and visfatin [11.93 (6.13–16.72) ng/ml vs. 13.01 (9.46–27.54) ng/ml, *P* < 0.001] than control counterparts, while the two study groups had non-significant differences between apelin and RBP-4 levels (shown in Figure 2). Differences between sECAS and NCAS included gender, HDL levels, and the levels of apelin and visfatin (*P* < 0.0125, Bonferroni correction). There were more male patients in sECAS than sICAS. The prevalence of traditional vascular risk factors and laboratory biomarkers showed no differences between the two groups. However, NIHSS at admission and mRS at discharge of sECAS were lower than sICAS. As for the four novel adipokines analyzed in our study, median plasma concentrations of apelin were lower in the sECAS (84.94, 46.88–130.41 ng/mL) than in the sICAS (118.64, 93.22–145.08 ng/mL) group (*P* = 0.002) (shown in Figure 2). However, levels of omentin, visfatin, and RBP-4 showed no significant differences between these two groups. This data shows that lower levels of apelin seem to be more associated with sECAS.

**Figure 4:**
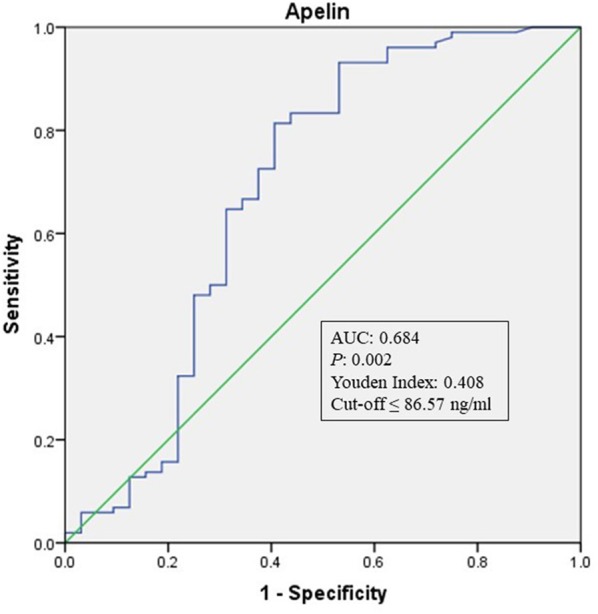
ROC curve analysis of Apelin cutoff point for the presence of sECAS (vs. sICAS). AUC, area under the curve; ROC, receiver operating characteristic; sICAS, symptomatic intracranial atherosclerotic stenosis disease; sECAS, symptomatic extracranial atherosclerotic stenosis disease.

To explore whether apelin could differentiate sECAS from sICAS, we performed logistic regression analysis. It showed that the lowermost tertile of apelin was independently associated with sECAS (OR, 5.121; 95% CI, 1.597–16.426) when adjusted for age, gender, hypertension, diabetes, dyslipidemia, MetS, and NIHSS at admission (as shown in Table 3). In ROC curves analysis, apelin had reasonable accuracy for prediction of sECAS (AUC: 0.684, Youden Index: 0.408, *P* = 0.002). The optimal cut-off value of omentin was ≤ 86.57 ng/mL (sensitivity: 0.814; specificity: 0.594) (as Figure 4 showed).

**Table 3:** Logistic regression analysis with the presence of sECAS as the dependent variable.

	**sECAS vs. sICAS**		
	***P-*value**	**OR**	**95% CI for OR**
Age	0.605	1.013	0.965–1.063
Gender (Male vs. Female)	0.051	3.101	0.993–9.684
Hypertension	0.336	0.563	0.175–1.814
Diabetes	0.244	2.094	0.604–7.262
Dyslipidemia	0.116	0.463	0.177–1.211
MetS	0.411	0.604	0.182–2.009
NIHSS on admission	0.047	0.881	0.777–0.998
Apelin tertiles, ng/ml			
1 (<91.14)	0.006	5.121	1.597–16.426
2 (91.14–132.56)	0.296	0.505	0.140–1.818
3 (>132.56)	Reference	-	-
*P* for trend = 0.001			

*sICAS, symptomatic intracranial atherosclerotic stenosis disease; sECAS, symptomatic extracranial atherosclerotic stenosis disease; Mets, metabolic syndrome; NIHSS, the National Institutes of Health Stroke Scale. sICAS is as reference*.

### Adipokines and the Severity of sICAS

Among 102 patients with sICAS, 27 (26.5%) patients had moderate stenosis and 75 (73.5%) had severe stenosis. Additionally, according to the number of stenotic vessels, 30 patients (29.4%) had single sICAS, 30 (29.4%) had two affected vessels and 42 (41.2%) had multi-stenoses. Spearman coefficient analysis showed no significant association between the four adipokines and the severity of intracranial atherosclerosis or the number of vessels with intracranial stenoses (data not shown).

### Stroke Severity and Short-term Prognosis of sICAS

Patients with severe stroke had lower levels of apelin (*P* = 0.005); the other three adipokines showed no such differences. Individuals with unfavorable outcome in the acute phase (14 days) of sICAS also showed no difference between these four adipokines when compared with the favorable group (shown in Table 4).

**Table 4:** Associations between novel adipokines and stroke severity or short-term prognosis of sICAS group.

	**Stroke severity**			**Short-term prognosis**		
	**NIHSS ≤8** **(*n* = 78)**	**NIHSS >8** **(*n* = 24)**	***P-*value**	**mRS ≤2** **(*n* = 52)**	**mRS >2** **(*n* = 50)**	***P-*value**
Apelin (ng/ml)	128.31 (100.41–148.36)	106.25 (68.59–123.39)	0.005	128.50 (101.08–155.16)	115.25 (86.64–131.76)	0.053
Omentin (ng/ml)	39.10 (30.98–50.70)	41.19 (28.74–59.41)	0.972	39.47 (32.03–51.55)	39.38 (28.78–49.75)	0.512
Visfatin (ng/ml)	12.27 (6.02–17.28)	10.80 (6.65–15.81)	0.795	12.76 (5.17–17.12)	10.78 (6.15–16.55)	0.781
RBP-4 (mg/L)	70.08 (48.46–109.08)	81.48 (61.16–121.03)	0.188	67.48 (48.05–115.69)	80.81 (53.99–107.64)	0.549

*sICAS, symptomatic intracranial atherosclerotic stenosis disease; NIHSS, the National Institutes of Health Stroke Scale; mRS, the modified Rankins Score*.

### Mortality and Long-Term Prognosis of sICAS

Long-term follow-ups of sICAS patients were conducted in the 12 months after discharge. During follow-up, a total of ten patients died, eight of whom died due to cerebrovascular reasons and two patients due to pneumonia. Seventy patients had favorable long-term outcome and 32 patients had unfavorable outcome. However, we found no differences of these four novel adipokines between groups (shown in Table 5).

**Table 5:** Associations between novel adipokines and long-term prognosis or mortality of sICAS group.

	**Long-term prognosis**			**Mortality**		
	**mRS ≤2** **(*n* = 70)**	**mRS >2** **(*n* =32)**	***P-*value**	**Survivors (*n* =92)**	**Non-survivors** **(*n* = 10)**	***P-*value**
Apelin	118.92 (93.22–148.37)	118.64 (90.73–131.53)	0.486	121.07 (91.57–146.38)	116.48 (88.74–128.60)	0.621
Omentin	37.31 (29.48–51.76)	42.81 (30.02–50.76)	0.562	39.43 (30.47–50.40)	42.35 (28.36–59.46)	0.941
Visfatin	11.93 (6.02–16.72)	11.97 (6.78–18.03)	0.599	11.93 (6.11–17.66)	11.65 (8.92–16.14)	1.000
RBP-4	80.81 (53.13–116.40)	70.82 (46.12–97.75)	0.313	76.07 (51.86–113.97)	76.36 (51.74–93.51)	0.812

*sICAS, symptomatic intracranial atherosclerotic stenosis disease; mRS, the modified Rankins Score*.

## Discussion

Our study showed that admission plasma omentin levels were significantly decreased in LAA patients when compared with NCAS subjects. Furthermore, lower admission plasma apelin levels were identified as reliable and independent markers to predict patients with sECAS, substantiating their potential role as a new diagnostic biomarker. However, we failed to find the prognostic values of these novel adipokines for short- and long-term outcomes and sICAS mortality. To our knowledge, this is the first study to assess the diagnostic and prognostic relationship of novel adipokines, apelin, omentin, visfatin, and RBP-4 with sICAS and sECAS.

In our study, LAA risk increased when levels of omentin decreased, supporting a potential protective role of omentin. As an anti-inflammatory adipokine, decreased levels of omentin-1 have been found in obesity, DM and MetS (40, 41). Previous researchers have found a negative correlation between omentin-1 levels and carotid atherosclerosis. Yoo et al. proved that low levels of omentin-1 were an independent factor of carotid plaque existence among patients with type 2 DM (42). The study by Kadoglou et al. demonstrated that patients with carotid atherosclerosis had lower omentin-1 levels when compared with patients without atherosclerosis (24). Our study adds evidence to the protective role of omentin by demonstrating that patients with LAA had decreased omentin-1 levels when compared to NCAS. The possible mechanisms underlying the protective effect of omentin in LAA can be summarized as follows: (1) omentin attenuates vascular inflammation through a variety of signaling pathways like the AMPK pathway. Research shows that through the AMPK signaling pathway, omentin deficiency can accelerate the formation of atherosclerotic plaque by promoting inflammatory response (2, 41) Omentin promotes angiogenesis by activating eNOS-dependent signaling pathway and enhances vascular dilatation of vascular endothelial cells in response to ischemia (3, 43, 44) omentin can improves insulin sensitivity by activating the Akt protein kinase B in adiposity as it increases the insulin transduction (45). Reduced insulin resistance and inflammation response caused by omentin may resist the formation of atherogenesis, and as a result, lower the risk of LAA.

Intracranial vessels differ from extracranial vessels by having a high antioxidant capacity, low inflammatory response, and a lack of vasa vasorum. They are also surrounded by cerebrospinal fluid (46). As we described in a recent paper (7), lower levels of vascular endothelial growth factor were more correlated with the presence of sICAS than sECAS, which suggests that angiogenesis in sICAS might be suppressed. In this study, lower apelin plasma levels are more associated with sECAS than with sICAS, and sICAS patients with severe stroke had lower levels of apelin. The role of apelin in the development of atherosclerotic diseases is not entirely clear. One study has demonstrated lower apelin levels in patients with carotid atherosclerosis. Since plaque vulnerability has been shown to correlate with an increase local number of inflammatory cells within plaques, a possible underlying mechanism could be that apelin limits macrophage infiltration (47). Apelin is also involved in the development of angiogenesis. It can promote angiogenesis response and cerebral reperfusion through the vascular endothelial growth factor-vascular endothelial growth factor receptor 2 (VEGF-VEGFR2) signaling pathway (48). In acute phase of sICAS, as a “good” adipokine, apelin can facilitate the angiogenesis reaction to increased oxygen supply. On the other hands, the lower levels of apelin means poor collateral circulations, causing neurological deficit symptoms to worsern.

In this study, we failed to discover the relationship between these novel adipokines and short- or long-term outcome of sICAS. This could be due to the fact that adipokines levels were only measured once at admission.

There are also some limitations in our research. Firstly, this study was a single- center cohort, and the sample size was relatively small. The external validity of the findings is limited. Secondly, adipokines levels in our study were only measured once on admission, as discussed above. Furthermore, other novel adipokines, such as vaspin, ghrelin, were not detected in our study.

## Conclusion

In conclusion, our present study demonstrated that lower levels of omentin are an independent biomarker of LAA while lower apelin plasma levels are more associated with sECAS. Omentin, apelin, visfatin, and RBP-4 had no prognostic value of sICAS. Our findings may be useful in assessing different atherosclerotic stenosis, detecting possible mechanisms of stroke, and may be of practical significance in the study of specific prevention strategies for sICAS and sECAS.

## Data Availability Statement

The datasets generated for this study are available on request to the corresponding author.

## Ethics Statement

All procedures performed in studies involving human participants were in accordance with the ethical standards of the institutional and/or national research committee and with the 1964 Helsinki declaration and its later amendments or comparable ethical standards. All subjects have signed the informed consent and the study protocol was approved by the Ethics Committee of Xiangya Hospital of the Central South University in China.

## Author Contributions

JX, BX, and QY involved in the study design. XZ, ZLi, and XF were responsible for the data collection. ZLiu, DL, QH, and XL were responsible for the sample collection. FY conducted the experiments and wrote the manuscript. JX modified and revised the manuscript. All authors have read and approved the final version of the manuscript.

### Conflict of Interest

The authors declare that this study received funding from Clinical and rehabilitation research fund of Sinobioway Biomedicine Co., Ltd.
